# Measurement Issues in Anthropometric Measures of Limb Volume Change in Persons at Risk for and Living with Lymphedema: A Reliability Study

**DOI:** 10.3390/jpm5040341

**Published:** 2015-09-30

**Authors:** Dorit Tidhar, Jane M. Armer, Daniel Deutscher, Chi-Ren Shyu, Josef Azuri, Richard Madsen

**Affiliations:** 1 Physical Therapy Service, Maccabi Healthcare Services, Tel-Aviv 6812511, Israel; E-Mail: Tidhar_d@mac.org.il; 2 Sinclair School of Nursing, University of Missouri, Columbia, MO 65211, USA; E-Mail: armer@missouri.edu; 3 Department of Computer Science, Informatics Institute, University of Missouri, Columbia, MO 65211, USA; E-Mail: shyuc@missouri.edu; 4 Health Division, Maccabi Healthcare Services, Tel-Aviv 6812511, Israel; E-Mail: azuri_yo@mac.org.il; 5 Sackler Faculty of Medicine, Tel-Aviv University, Tel-Aviv 69978, Israel; 6 Office of Medical Research, University of Missouri, Columbia, MO 65212, USA; E-Mail: MadsenR@missouri.edu

**Keywords:** decision-making, lymphedema, lymphedema management, minimal clinical, detectable change, reliability, standard error of measurement, tape measurement

## Abstract

Understanding whether a true change has occurred during the process of care is of utmost importance in lymphedema management secondary to cancer treatments. Decisions about when to order a garment, start an exercise program, and begin or end therapy are based primarily on measurements of limb volume, based on circumferences taken by physiotherapists using a flexible tape. This study aimed to assess intra-rater and inter-rater reliability of measurements taken by physiotherapists of legs and arms with and without lymphedema and to evaluate whether there is a difference in reliability when measuring a healthy *versus* a lymphedematous limb. The intra-rater reliability of arm and leg measurements by trained physiotherapist were very high (scaled standard error of measurements (*SEM*s) for an arm and a leg volume were 0.82% and 0.64%, respectively) and a cut-point of 1% scaled *SEM* may be recommended as a threshold for acceptable reliability. Physiotherapists can rely on the same error when assessing lymphedematous or healthy limbs. For those who work in teams and share patients, practice is needed in synchronizing the measurements and regularly monitoring their inter-rater reliability.

## 1. Introduction

Post-cancer treatments lymphedema (LE) is a chronic disease, which has no cure [1]; however, it can be managed successfully by reducing symptoms and volume and maintaining that reduction [2]. LE early detection is performed by measuring volume and assessing symptoms and comparing them to baseline measurements [3]. Management strategies vary and may include, for example, daily bandaging, performing exercises twice weekly, using a pneumatic compression device, or receiving manual lymph drainage. As long as patients improve by reaching their treatment goals, the treatment will be considered a success [4]. Examples of treatment goals may be: improving a specific function, reducing symptoms such as pain or heaviness, preventing infection, improving body image, improving limb shape, *etc*. Assessing LE outcomes only from the patient’s perspective is not sufficient for therapists in their decision-making process; objective assessment, such as volume measurement, is needed as well. Therefore, when resources are limited, and the question of adherence to treatment is considered physiotherapists (PTs) are interested in offering patients the most efficient tool or technique, one that will achieve successful outcomes.

One of the main outcomes for LE treatment is volume reduction that can be measured using different approaches. The Perometer™ is a device that uses infrared light beams to estimate the volume of a limb [5]. Water displacement is used to assess the volume of a limb by submerging the arm or leg in a water tank and measuring the water that is displaced. This method has been considered to be the “gold standard” [6]. A flexible measurement tape is a practical low-cost tool available in any clinic. Volume is derived from calculations of several circumferential measurements taken at predetermined points along the limb using a truncated cone formula [7].

Knowing the minimum clinically important difference (MCID) of limb volume change is essential for the clinician in determining the existence of LE, its improvement, progression, or stability [4]. There are numerous possible consequences to that decision. A garment ordered too soon may not fit and, therefore, result in a financial loss. Requiring a patient to wait longer than necessary and continue treatments may lead to reduced adherence to attend the therapy sessions as she/he may feel stable and resent wasting time and money going to therapy. Prolonging treatment beyond what is needed could increase patient waiting lists to the detriment of lymphedema management services. When assessing patients’ progress using the flexible tape method, the standard error of measurement (*SEM*) may be used by clinicians as a Distribution based MCID [8]. In a case series by Tidhar *et al.* [9], the clinical decision-making to order a garment was based on volume stability. The authors reported a *SEM* of 30 mL for the therapist treating patients in a self-bandaging clinic, a threshold below which was considered stable and beyond which was considered unstable [9]. Schmidt *et al.* [10] defined the term “stable lymphedema” to help determine whether a woman with breast cancer-related LE (BCRL) could begin a weight-lifting program; one criterion was that the woman experience no more than 10% increase in arm swelling in the three preceding months. PTs who want to implement this weight-lifting program and who must decide whether to approve an individual’s participation need to know their own scaled *SEM* in order to determine whether their patients are stable or not.

Few studies have examined the Distribution based MCID of limb volume change: Katz Leurer *et al.* [11] reported *SEM* of 78.8 mL of the healthy arm *vs.* 47.1 mL of the affected arm in BCRL, Taylor *et al.* [7] found the *SEM* of BCRL to be in a range of 64.5–65.4 mL [7], and Devoogt *et al.* [12] reported a minimal detectable change (MDC) of 55 mL to detect a true change. One study by Sawan *et al.* [13] reported a repeatability limit of 270 mL for leg volume; no other reports were found for *SEM* of leg volume. Devoogt *et al.* [12] reported on scaled *SEM* that ranged from 0.8%–2% for measuring arms; no report was found on scaled *SEM* for leg LE measurement.

Sometimes PTs work together in the same clinic and share patient management. Knowing the inter-rater reliability of volume measurements is most important if they want to base their decisions on their colleague's assessment. Several studies have examined the inter-rater reliability in measuring arm volume and reported an intra-class correlation (ICC) of more than 0.97 between raters [7,14]. Unfortunately, ICC cannot be translated to clinical practice as the dimensions are different from those used in practice. Sawan *et al.* (2009) reported on inter-rater reliability and measured reproducibility of 1000 mL when measuring one leg volume by 17 assessors. These findings have not been supported by any other study.

The aims of this study were: (a) to determine the *SEM* for measuring volume of an arm and a leg with and without LE for the purpose of assessing and following up on patients with LE and surveying those who have not yet developed LE; (b) to assess if there is a difference between *SEM* of healthy *vs.* LE limbs; and (c) to examine the reliability between therapists when measuring an arm and a leg with and without LE.

## 2. Design and Methods

Sixty PTs who work in lymphedema management in Maccabi Healthcare Services and in private practice, received an invitation to attend the day of research, which contained the purpose and schedule of the day. Forty-one PTs volunteered and participated in the study that took place as part of a conference organized by the physiotherapy department of Maccabi Healthcare Services in Israel. All PTs were lymphedema specialists who had received 140 h of training and had more than one year’s experience in measuring patients’ limb volume (range from 1 to 20 years). This study was conducted as part of a practice-based evidence research process in which we test the reliability of PT’s documentation before we start collecting data. In all, 41 PTs attend and volunteered to participate. The study was approved by the Ethics committee of Maccabi Healthcare Services.

Five patients were invited to participate in the study. Patients were asked to be present half an hour prior to the taking of measurements so they could lie supine to reduce any excess fluid that might have accumulated from commuting to the study venue. Prior to participation, consent was obtained from all patients. At the beginning of the conference, the primary author (DT) gave a short demonstration of the measuring procedure. Each physiotherapist (PT) left the conference for approximately 20 min throughout the day and measured one patient. Each patient volunteered his/her LE limb and a healthy limb (e.g., a woman with arm LE had her lymphedematous arm and a healthy leg measured). Both limbs were measured 3 times.

Circumferential measurements were taken at 6 points on an arm according to anatomical landmarks [7] (mid palm, wrist, 10 cm above the wrist point, elbow, 10 cm above elbow and axilla), and 8 measurements for a leg using a measurement board (10 cm from heel towards toes, 10 cm from heel towards ankle, 20 cm, 30 cm, 40 cm, knee, 55 cm and Groin).

The circumferences were then entered into a spreadsheet and a truncated cone formula applied with each segment volume calculated:

(1) $$Vs=\;\frac{h\left(Ct\times Ct+Ct\times Cb+Cb\times Cb\right)}{12\pi }$$

where *Vs.* was volume of a segment, *h* was the distance between two points of measurement, *Ct* represented the circumference at the top measurements of the segment, *Cb* represented the circumference at the base of the segment. Once each segment was calculated, a sum of five segments of an arm and seven segments of the leg were computed into a volume estimate [15]. This method was found to be valid (criterion validity) in several studies when compared to the gold standard of water displacement with intra class correlation coefficient (ICC) of >0.95 [7,16].

## 3. Data Analysis

*SEM* and Scaled *SEM* of limb volume were obtained by using the Formulas (2) and (3):

(2) $$SEM=\;\frac{s}{\sqrt{3}}$$

where *s* as the standard deviation of 3 volume measurements of one PT.

Scaled *SEM* was calculated by the formula:

(3) $$Scaled\;\left(\%\right)SEM=\;\frac{SEM}{\bar{v}}\times 100$$

Where the *SEM* is divided by the average of volume and multiplied by 100 to create a standard estimate that can be compared between leg and arm measurements. The data of both *SEM* and scaled *SEM* is presented as an average and confidence interval (CI) (for all PTs). As the definition for early detection of BCRL has been proposed as 3% difference from baseline [17], a 1% scaled *SEM* seemed like an appropriate cutoff point for clinical use; therefore, the proportion of PTs whose scaled *SEM* did not exceed the 1% was calculated. Differences of scaled *SEM* between the groups were analyzed by Kruskal-Wallis nonparametric test to check whether one patient was more difficult to measure than others, resulting in significantly different outcome.

For analysis of the quality of the limb volume measurements made by different PTs, we used the difference between their first measurement from the true mean (which was defined as the average of all measurements by all therapists of the same limb) scaled by the true mean (Formula (4)). We then estimated the proportion of therapists whose measurements were within 5% of the true mean. As 10% difference between limbs is considered one of the definitions for LE [18,19], and was determined as defining stable LE as well [20], and while recognizing that the choice is arbitrary, a 5% limit within the true mean was chosen to be an appropriate cutoff point for a team of PTs measuring the same patient.

The percentage difference (% diff) from the true mean for a single PT was calculated using the formula:

(4) $$\text{\%diff}=\;\frac{v_{1}-\bar{v}}{\bar{v}}\times 100$$

where $v_{1}$ is the first volume measurement (out of three), $\bar{v}\;$ is the average of the volumes from all PTs who measured the same limb (only the first volume measurement was used since in clinical practice usually only one measurement is taken).

Differences between measurements from healthy and lymphedemadematous limbs were analyzed by Wilcoxon Rank Sum test. IBM SPSS software version 21 was used to analyze all data.

## 4. Results

Five patients with secondary lymphedema participated in the study. Two were women with upper extremity LE following breast cancer, one was a woman with lower limb LE following treatment for sarcoma and two were men with phlebolymphedema of the lower extremity. The limbs to be measured included three healthy arms and two with LE (one severe and one mild), and two healthy legs and three with LE (one moderate and two severe). Forty-one PTs were divided into five groups of 6–11 each.

Aim 1: Intera-rater reliability.

**Figure 1:**
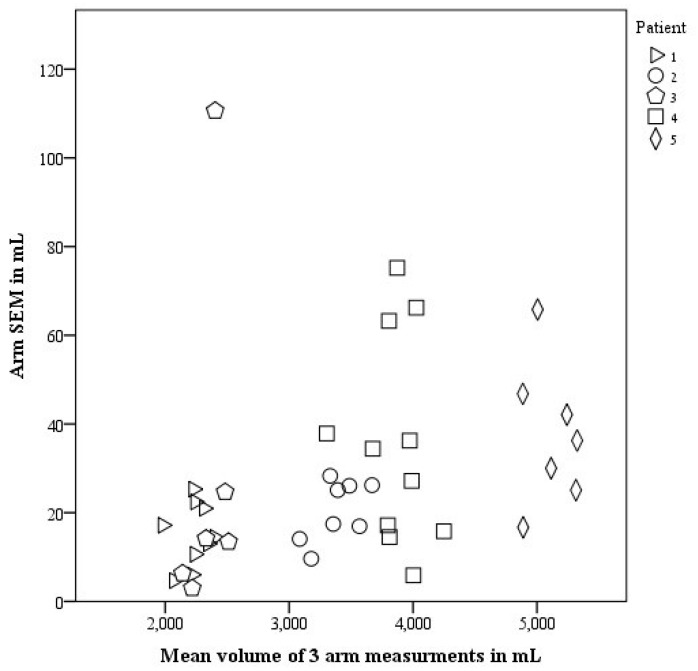
*SEM* in mL for 41 PTs measuring an arm; each symbol represents a patient.

**Figure 2:**
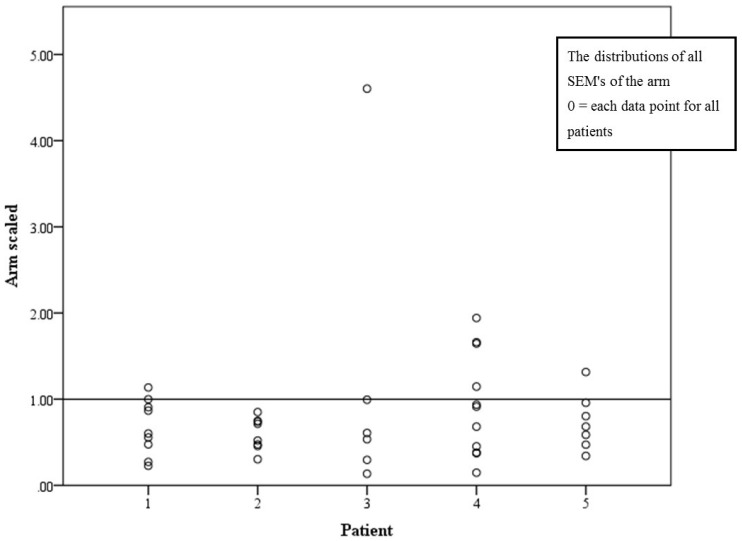
Scaled *SEM* presented in percentage, with the line at 1% cutoff.

Average *SEM* for arm measurements was 27.5 mL (CI 20.5–34.4 mL). Figure 1 demonstrates the distributions of all *SEM’*s of the arm. Mean scaled *SEM* was 0.82% (CI 0.59%–1.05%). The proportion of PTs with scaled *SEM* less than 1% was 83% (Figure 2). From both Figure 1 and Figure 2, an outlier is obvious with a *SEM* of 110 mL and scaled *SEM* of 4.6%. Since all other PTs who had measured the same patient’s arm had scaled *SEM* below 1%, it is obvious that this PT’s technique requires improvement. The group who measured patient 4 seemed to have fewer PTs who measured within the 1% cutoff point; however, when analyzing the differences between groups, no statistical differences were found in the scaled *SEM* (*p* = 0.847).

**Figure 3:**
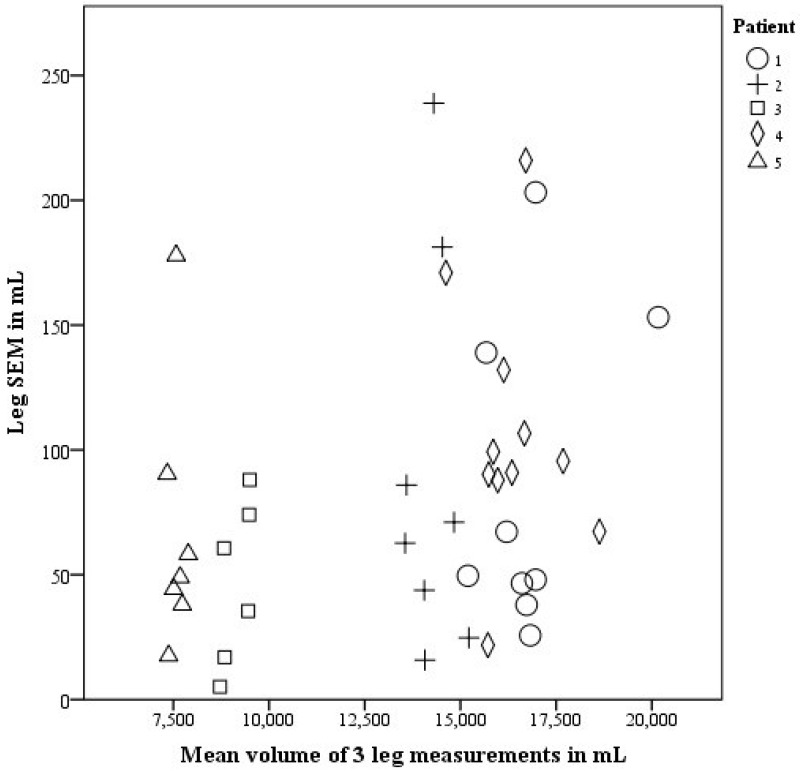
*SEM* in mL for 41 PTs measuring a leg, each symbol represents a patient.

Average *SEM* for leg measurements was 83.6 mL (CI 65–102 mL). Figure 3 demonstrates the distributions of all *SEM’*s of the leg.

**Figure 4:**
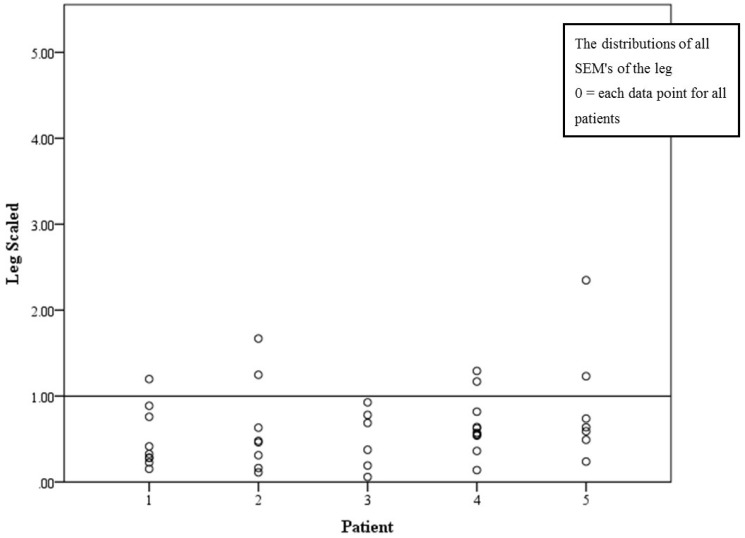
Scaled *SEM* presented in percentage, with the line at 1% cutoff.

Scaled *SEM* was 0.64% *SEM* (CI 0.5%–0.78%). The proportion of PTs whose scaled *SEM* was less than 1% was 83% (34/41) (Figure 4). There was no statistically significant difference of scaled *SEM* of legs (*p* = 0.598) between the groups measuring different patients.

Aim 2: *SEM* of healthy *vs.* lymphedema.

There was no statistically significant difference between scaled *SEM* for healthy *vs.* LE arms (*p* = 0.945) or for legs (*p* = 0.533).

Aim 3: Inter-rater reliability.

**Figure 5:**
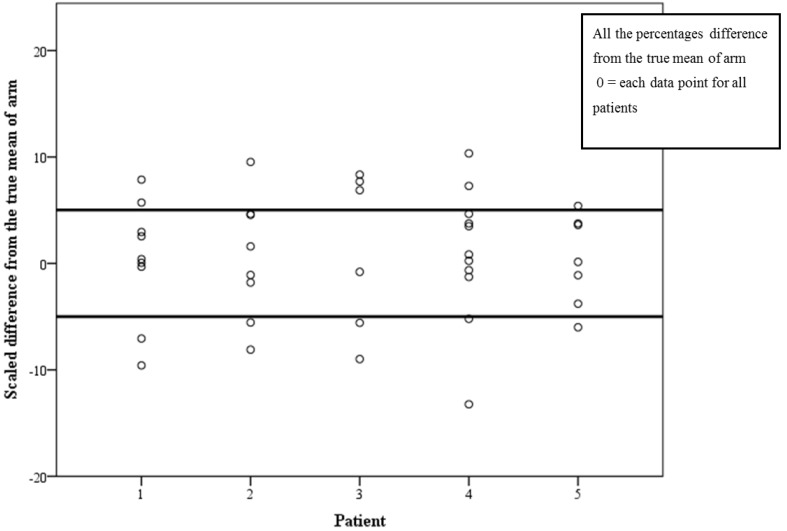
Scaled difference from the true mean of arm measurements for each PT with cutoff points at ± 5%.

**Figure 6:**
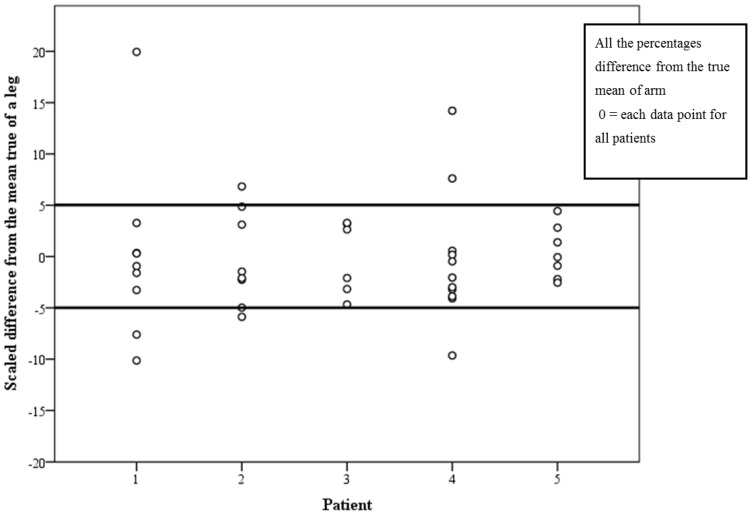
Scaled difference from the true mean of leg measurements for each PT with cutoff points at ± 5%.

Fifty-six percent (23/41) of PTs’ first measurement was within 5% of the true mean value for measuring an arm with CI of 42%–72% (Figure 5) and 80.5% (33/41) of PTs’ first measurement was within 5% of the true mean value for measuring a leg with CI of 68%–93% (Figure 6).

## 5. Discussion

The first purpose of the study (Aim 1) was to examine intra-rater reliability of limb volume measurement for each PT. We found a mean *SEM* for an arm to be 27.5 mL which is consistent with the study by Tidhar *et al.* [21], who reported 30 mL *SEM*, and with Bracha *et al.* [22] who reported 26.2 and 23.2 mL for two raters in their study. For the leg, we found a mean *SEM* of 83.6 mL. The only study which reported intra-rater reliability of leg measurement was performed by Sawan *et al.* [13] and found a repeatability limit of 270 mL; however, the *SEM* was not reported which makes it difficult to compare to our findings. In our study, the mean scaled *SEM* for arm was 0.82%, which is consistent with Devoogt *et al.*’s [12] report of scaled *SEM* that ranged from 0.8%–2%. The mean scaled *SEM* for leg was found to be 0.64% in our study; however, no report was found on scaled *SEM* for leg LE measurement in other studies. Since the majority (83%) of PTs measured below the 1% scaled *SEM* and as no recommendation regarding MCID for scaled *SEM* exists in the literature for legs or for arms, we recommend that a Distribution based MCID for scaled *SEM* will be set at 1% by clinicians when assessing both arm or leg LE.

Assessing whether there is a difference between scaled *SEM* of healthy *vs.* lymphedema limbs was the second purpose of this study (Aim 2). We did not find any statistically significant difference in the scaled *SEM* between healthy *vs.* affected arm or leg. Katz Leurer *et al.* [11] found different *SEM* for healthy *vs.* LE arms; however, they did not report whether these findings were significant. This information is important since the PTs who participated in our study now know that they may use the same scaled *SEM* when comparing affected limb to healthy one in detecting LE and as part of assessments and follow-ups in treatment routine.

The third aim of this study (Aim 3) was to examine the reliability between therapists when measuring an arm and a leg with and without LE. In most reliability studies the protocol was for two measurers to assess several subjects; in our study, 41 PTs each measured one arm and one leg. This design strengthens the external validity of the study, as we have 41 PTs who work in lymphedema services all over the country. Furthermore, standardization of measurement technique is important for routine clinical documentation. The purpose of examining the inter-rater reliability is to ensure PTs who treat the same patients can rely on each other’s outcomes. We examined the difference between the PT first measurements from the true mean and found that 56% and 80.5% of PT’s who assessed an arm and a leg, respectively, measured within the ±5% range. No reported studies have used this method of assessing inter-rater reliability in limb volume measurement. However, we propose this method to be used clinically as it is a practical way of assessing whether a group of PTs can work together to achieve high inter-rater reliability (e.g., if a PT got a score of 7% it means that he/she may have trouble relying on their colleague’s measurements when they share a patient management). If all PTs in a team obtained scores within the ±5% range, sharing patients’ care can become more practical and reliable.

Results of the arm measurements from this study are not acceptable for clinical practice. They may be explained by the fact that the points of measurements were not constant; the PTs were instructed to measure at anatomical landmarks which are easy to detect, but were not marked on the arms. Therefore, although the *SEM* that represents the consistency of an individual PT was very good, the agreement between 41 PTs was not. For the leg measurements, the instructions were to use a measurement board which enabled more constant points and, therefore, the higher scores. Most of our PTs work alone in a clinic; however, for those who do work as a team, we recommend practicing their measurements with feedback from the person who is being measured for consistency of tape tension and points of measuring. Although not reported in the literature, from clinical experience, this is a useful way of improving consistency. When measuring an arm, a measuring board may be used to improve the inter-rater reliability.

## 6. Limitations

The study has a few limitations regarding the design. As we conducted the study as part of a conference day, it was not practical that all PTs measured all patients or even one patient. Therefore, we chose the design of dividing PTs into five groups and for each PT in a group to measure the same patient with the risk of finding differences in *SEM* and scaled *SEM* between limbs, at the cost of increased patient burden; however, no differences were found in the scaled *SEM* of the arm and leg between limbs. Nevertheless, having PTs measure all patients would have increased the external validity of the study.

The results of the inter-rater reliability of the arm measurements raise another limitation. As only 56% of PTs measured within the limit of ±5% from the true mean, we question whether we could have changed the design of the study to prevent this from occurring. We could have expected that 41 PTs would not measure within the same inter-rater limit, even though intra-rater measurements were very constant. Usually lymphedema therapy is performed by a single PT; however, in some clinics there are teams of PTs who share patients’ care. Therefore, we could have assigned PTs to their clinical teams, where possible, instead of randomly dividing them to groups. This way we would be able to provide a clinical tool for team work; however, this can still be achieved in clinical practice setting as well.

## 7. Conclusions

This study examined the intra- and inter-rater reliability of PTs measuring both arms and legs of patients with lymphedema. *SEM* and scaled *SEM* were analyzed and appear to be consistent with the literature with a mean *SEM* of 27.5 mL and 0.82% scaled *SEM* for an arm and a mean *SEM* of 83.6 mL and 0.64% scaled *SEM* for a leg. Most of the PTs measured below the 1% scaled *SEM*; therefore, the authors recommend that a 1% scaled *SEM* should be considered a threshold (MCID) of an arm and leg measurements; however, an anchor-based approach needs to be verified to assess whether this threshold is clinically meaningful as well. Our findings show that there was no difference between scaled *SEM* of healthy *vs.* LE limbs; therefore, we suggest that the same scaled *SEM* can be used when measuring a healthy or LE limbs.

Furthermore, at the end of the analyses, a personal letter was sent to all PTs with information about their own *SEM* and scaled *SEM*. The PTs were encouraged to use their own *SEM* and scaled *SEM* as part of their practical reasoning process when treating patients with lymphedema, as was demonstrated in the study by Tidhar *et al.* [9] regarding decisions that were made for ordering a garment. Deyes *et al.* [23] reported a cost of $1500 for intensive therapy of 20 sessions in Canada. Stout *et al.* [24] reported on 15 sessions, l which cost $3000 in the USA. Using a personal *SEM* to detect when the patient’s limb reaches a plateau may improve the cost effectiveness of LE treatments by optimizing the number of visits provided to the patient’s in accordance with the significance of limb volume changes between visits. Beyond the purpose of this study, the anatomical landmarks method is being implemented for documentation in the clinical medical records in Maccabi Healthcare Services physiotherapy clinics. Therefore, PTs who are not accustomed to this method of measurement need to practice more to increase their confidence and reliability in the clinical protocols. Hence, PTs who exceeded the 1% scaled *SEM* were recommended to practice and test their scaled *SEM* further until they reach the cutoff point of 1% or below.

When working in the same clinic as a team, PTs who measure the same patients should practice together to reach acceptable agreement in their *SEM*. When a clinician needs to make decisions that involve resources such as time, money, adherence, and motivation, having a tool that will increase confidence in the decision-making process is important. *SEM* is such a tool.
